# Supporting patients in the transition to the revised pexidartinib dosing regimen: perspectives from the multidisciplinary clinical and allied health professional team

**DOI:** 10.1186/s13023-023-02926-9

**Published:** 2023-10-07

**Authors:** Colleen McCabe, Hillary Wright, Kathleen Polson, Andrew J. Wagner

**Affiliations:** 1 Department of Pharmaceutical Services, Vanderbilt University Medical Center, Nashville, TN USA; 2 Director for Nutrition Counseling, Wellness Center at Boston IVF, Waltham, MA USA; 3 Dana-Farber Cancer Institute, Center for Sarcoma and Bone Oncology, Boston, MA USA

**Keywords:** Pexidartinib, Label change, Dosing, Dosing regimen, Administration, Fasted, Low-fat meal, TGCT, Tenosynovial giant cell tumor

## Abstract

Pexidartinib is a colony-stimulating factor-1 receptor inhibitor approved in the United States for treatment of adult patients with symptomatic tenosynovial giant cell tumor (TGCT) associated with severe morbidity or functional limitations and not amenable to improvement with surgery. Because of the risk of severe and potentially fatal hepatotoxicity, pexidartinib is only available through a Risk Evaluation and Mitigation Strategy (REMS) program. Pexidartinib pharmacokinetics are influenced by the fat content of meals: compared with the fasted state, consuming a high-fat meal with pexidartinib increases pexidartinib absorption by 100%; a low-fat meal increases absorption by approximately 60%. Pexidartinib was initially authorized to be taken at 800 mg/day on an empty stomach; therefore, if this same dose of pexidartinib is taken with food, there is a risk of overexposure and potential toxicity. To reduce the risk of hepatotoxicity and improve patient compliance, pexidartinib has undergone a revised dosing regimen, from 800 mg/day (400 mg twice daily) fasted to 500 mg/day (250 mg twice daily) with a low-fat meal (approximately 11–14 g of total fat). The objective of this report is to educate clinical and allied health professionals on the revised dosing regimen and the importance of patient compliance with a low-fat meal. Healthcare professionals need to understand the rationale for the switch from pexidartinib dosing on an empty stomach to dosing with a low-fat meal and how meal composition and timing influence pharmacokinetics. Finally, we provide guidance for the healthcare team of prescribing providers, nurses, pharmacists, and dietitians who are caring for patients with TGCT on pexidartinib. It is important for healthcare providers to deliver consistent messaging on the low-fat meal requirement and help patients fit pexidartinib into their regular meal schedules. Consulting a dietitian may be helpful for patients, especially those with complex dietary needs. We provide an overview of the roles and responsibilities of each healthcare professional and outline steps to best support patients, including key questions and answers related to the revised dosing regimen. This report provides the information necessary to guide the multidisciplinary team caring for patients with TGCT and to support them through the pexidartinib dosing regimen change.

## Background

Pexidartinib is an orally administered small molecular tyrosine kinase inhibitor with strong activity against the colony-stimulated factor 1 receptor [1, 2]. It is approved by the US Food and Drug Administration (FDA) for the treatment of adult patients with symptomatic tenosynovial giant cell tumor (TGCT) associated with severe morbidity or functional limitations and not amenable to improvement with surgery [3]. Due to the risk of serious and potentially fatal hepatotoxicity, pexidartinib is only available through the Risk Evaluation and Mitigation Strategy (REMS) program [3]. Pharmacokinetic studies have shown that when pexidartinib was administered with a high-fat meal serum exposure approximately doubled [4]. Therefore, if patients take pexidartinib with a meal, overexposure can occur and possibly increase the risk of adverse events. Thus, a label update was sought to change pexidartinib dosing from administration at 800 mg/day (400 mg twice daily using 200-mg capsules) in a fasting state to administration with a low-fat meal at 500 mg/day (250 mg twice daily using 125-mg capsules). Although these potential benefits require further study, it is hypothesized that the revised dosing regimen will reduce the potential for drug overexposure and increase compliance with pexidartinib.

**Fig. 1:**
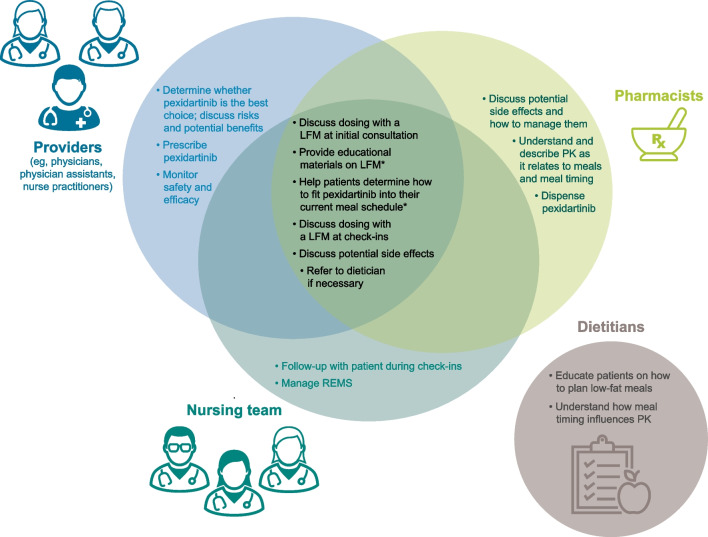
Roles within the multidisciplinary team. LFM, low-fat meal; PK, pharmacokinetics; REMS, Risk Evaluation and Mitigation Strategy. *Also included within dietitian sphere

TGCT can be a complex disease that requires a multidisciplinary team to ensure adequate care. This team is composed of the prescribing provider, nursing team, pharmacist, and dietitian. Each of these team members plays an important role in educating patients on the low-fat meal requirement, from deciding whether to take pexidartinib, to incorporating pexidartinib into patients’ daily schedules (Fig. 1). The objective of this report is to educate clinical and allied health professionals on the revised pexidartinib dosing regimen and the importance of patient compliance with the low-fat meal requirement. First, we review the rationale for the switch from pexidartinib dosing while fasted to dosing with a low-fat meal, discuss pharmacokinetics of pexidartinib as it relates to meal composition and timing, and finally review the role of each member of the multidisciplinary team and provide suggestions to help patients adhere to the new dosing regimen. We provide a perspective from our respective roles within our institutions; however, we acknowledge there are likely to be variations across institutions.

## Pexidartinib clinical data

Consistent messaging regarding dosing with a low-fat meal and what constitutes a low-fat meal is key to patient compliance; therefore, understanding of the clinical efficacy, safety, and pharmacokinetics of pexidartinib is essential for all members of the team.

### Efficacy and safety

Results of the phase 3 ENLIVEN study (ClinicalTrials.gov Identifier: NCT02371369) led to the approval of pexidartinib in the United States [2, 3]. ENLIVEN was a randomized, double-blind, placebo-controlled (part 1) then open-label (part 2), multinational trial [2]. Eligible patients were those with histologically confirmed, advanced, symptomatic TGCT ≥ 2 cm in size and measurable per Response Evaluation Criteria in Solid Tumors version 1.1 (RECIST) for whom surgical resection would be associated with a potential for worsening of functional limitation or severe morbidity. Patients in the pexidartinib group in part 1 received a loading dose of pexidartinib 1000 mg/day (400 mg in the morning, 600 mg in the evening) for the first 2 weeks, followed by 800 mg/day (400 mg twice daily) thereafter. At Week 25, the proportion of patients who achieved overall response was higher for pexidartinib (39%) than placebo (0%) as measured by RECIST. Response by tumor volume score was 56% in the pexidartinib group versus 0% in the placebo group. Patients’ relative range of motion, stiffness, and overall physical functioning were also improved with pexidartinib. The most common adverse events included hair color changes, fatigue, aspartate aminotransferase (AST)/alanine aminotransferase (ALT) increase, nausea, and dysgeusia [2]. Four (4%) patients from the ENLIVEN study experienced mixed or cholestatic hepatotoxicity; this resolved in all cases after stopping pexidartinib [5].

### Pharmacokinetic studies

The pharmacokinetics of pexidartinib have been studied in patients with TGCT and in healthy volunteers. The average half-life of pexidartinib is 26.6 h. In the fasted state, the median time to maximum plasma concentration (T_max_) is 2.5 h. Steady-state levels of pexidartinib are achieved after approximately 1 week [3].

Pharmacokinetic studies have examined the influence of high-fat and low-fat meals on pexidartinib absorption. Fat stimulates the release of bile and bile salts that increase pexidartinib absorption and consequently increase exposure [6]. Studies conducted in healthy volunteers showed that administering pexidartinib after a high-fat meal resulted in an approximate 100% increase in maximum plasma concentration (C_max_) compared with the fasted state for the 400-mg dose; absorption (area under the plasma concentration–time curve from time 0 to the last quantifiable concentration [AUC_last_] and area under the plasma concentration–time curve from time 0 extrapolated to infinity [AUC_inf_]) was similarly increased with a high-fat meal [4]. The T_max_ was also delayed with a high-fat meal compared to the fasted state. Additional studies demonstrated that this effect is maintained across doses of 400–1800 mg [4].

**Fig. 2:**
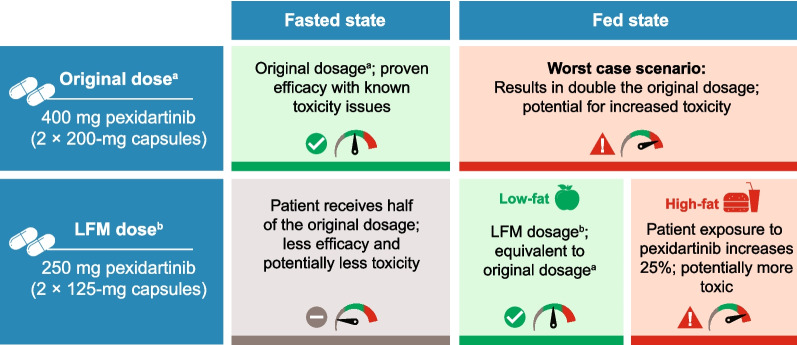
Changes in pexidartinib exposure after a high-fat or low-fat meal compared to the fasted state. LFM, low-fat meal. ^a^Lamb YN. Pexidartinib: first approval. Drugs. 2019;79(16):1805–1812. ^b^TURALIO^®^ (pexidartinib) capsules [package insert]. Basking Ridge, NJ: Daiichi Sankyo, Inc.; October 2022

Pharmacokinetic studies conducted in healthy volunteers revealed that administering pexidartinib with a low-fat meal increased exposure by approximately 60% relative to the fasted state [4]. When 400 mg pexidartinib was administered with a low-fat meal, there was a 56% increase in C_max_ and a 59% increase in AUC_inf_. Similarly, with 200 mg pexidartinib, there was a 71% increase in C_max_ and a 66% increase in AUC_inf_. Based on these results, it was hypothesized that administering 250 mg (2 × 125-mg capsules) pexidartinib twice daily with a low-fat meal (low-fat meal dosing regimen) would achieve an exposure similar to 400 mg administered during a fasted state and reduce the risk of inadvertent overdosage due to taking 400 mg with food. This was confirmed in a model assessment, which used clinical trial simulations to show that the prior dosing regimen (400 mg fasted twice daily) and the low-fat meal dosing regimen (250 mg with a low-fat meal twice daily) were predicted to yield similar exposures [4]. Therefore, these studies have demonstrated that the fat content of meals is an important consideration for pexidartinib absorption. Fat stimulates the release of bile and bile salts that increase pexidartinib absorption and consequently increase exposure [6]. The original and updated dosing information and effects on pharmacokinetics with a low- or high-fat meal is shown in Fig. 2.

A physiologically based pharmacokinetic (PBPK) model evaluated the impact of meal content and timing on pexidartinib exposure [7]. This study found that the fat content of a meal had a greater influence on pexidartinib exposure compared with calories, demonstrating the importance of controlling the fat content of meals. With respect to meal timing, a 15–16% increase in AUC_inf_ was predicted when consuming a high-fat meal 1 h after pexidartinib was taken with a low-fat meal, but a < 10% effect on PK was predicted when a high-fat meal was consumed ≥ 2 h after pexidartinib dosing with a low-fat meal. When a high-fat meal was consumed 2 h before pexidartinib dosing with a low-fat meal, exposure increased by 10–20%; however, a minimal effect was observed when a high-fat meal was consumed ≥ 3 h before pexidartinib was taken with a low-fat meal [7]. Therefore, when advising patients about which low-fat meals are suitable to be taken with pexidartinib, fat content is the most important consideration. The revised dosing regimen defines a low-fat meal as approximately 11–14 g of total fat [3]. When a high-fat meal is consumed, patients should wait ≥ 3 h before taking pexidartinib with a low-fat meal. After a patient takes their pexidartinib dose with a low-fat meal, they should wait ≥ 2 h before consuming a high-fat meal [7].

## Care is multidisciplinary

**Fig. 3:**
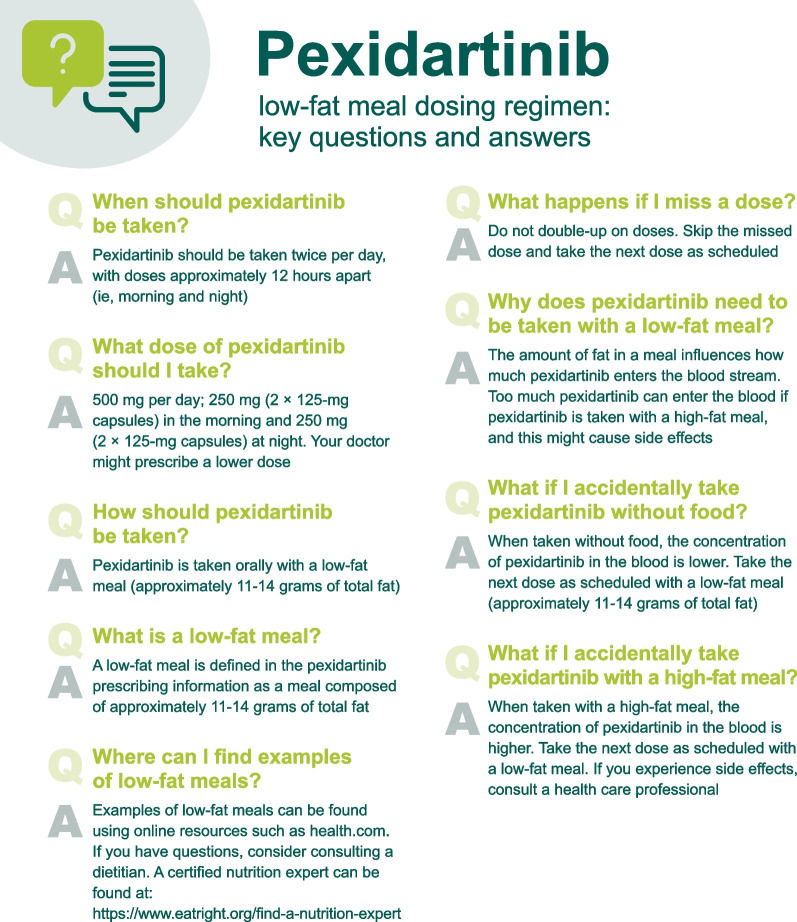
Key questions and answers related to the pexidartinib low-fat meal dosing regimen

Management of patients with TGCT generally involves a team where providers, pharmacists, and nurses are involved with the initial discussion, education, and short-term check-ins that are part of the REMS program, as well as long-term follow-up (Fig. 1). A dietitian can help provide support for patients with more complex meal requirements (e.g., those with food allergies, diabetes, gastric bypass, and/or weight loss struggles). Each of these team members will help support the patient when beginning pexidartinib or transitioning from the fasted to the low-fat meal dosing regimen. Because instructions are complex (e.g., grams of fat are not intuitive when meal planning), it is important to ensure that each team member delivers a consistent message with respect to dosing pexidartinib with a low-fat meal and that each team member is reviewing what patients are eating with their doses. The use of electronic or printed educational materials may be helpful for this aspect. Key questions and answers related to the pexidartinib low-fat meal dosing regimen are shown in Fig. 3.

### Provider’s perspective

As the individual responsible for prescribing pexidartinib, we begin with a provider’s perspective, which includes physicians, physician assistants, and nurse practitioners. During the consultation, there are several things for providers to consider. Even before writing a prescription, providers help patients decide whether pexidartinib is the best treatment option. Pexidartinib has been shown to significantly reduce tumor volume while also reducing signs and symptoms of TGCT for patients who have TGCT associated with severe morbidity or functional limitations and not amenable to surgery; however, pexidartinib is also associated with the risk of severe hepatotoxicity [3]. Therefore, the provider and patient must decide if the benefits outweigh any potential risks. In addition, prescribers must become certified in the pexidartinib REMS program to prescribe the therapy (turaliorems.com).

Providers should also inform patients that members of the nursing team and pharmacists will discuss pexidartinib administration as well as the details of the low-fat meal dosing requirement with them. For this aspect, it is helpful to have educational materials that provide the definition of “low-fat meal” per the FDA (approximately 11–14 g of total fat) [3, 8], samples of meals, steps to take in case of missed doses, and potential side effects. Key questions and answers related to the pexidartinib low-fat meal dosing regimen are shown in Fig. 3.

Providers are the initial point of contact and should review how to take pexidartinib, dosing, the low-fat meal requirement, and potential side effects. Due to the REMS requirements, providers and/or nurses will follow-up with patients regularly for the first 8 weeks, although which provider is responsible for these visits will vary across institutions. Patients who require additional monitoring due to the risk of poor tolerability may have more frequent check-ins. Generally, laboratory measurement of hepatic function will be monitored weekly. During these check-ins, the low-fat meal requirement can be reviewed. A patient’s location will influence the type of follow-up visit, which is more likely to be over the phone or in a virtual format if a patient must travel a long distance to get to the clinic; however, this is not a limitation as most of the discussion regarding the low-fat meal requirement can be conducted virtually.

While a valuable opportunity, meeting a dietitian might not be necessary or possible for all patients. Those patients with specific dietary requirements (due to special diets, food allergy, gastric bypass, and/or weight loss struggles) should have an initial consultation with a dietitian to ensure they are taking pexidartinib with a low-fat meal. Because there is often flexibility in the timing of starting treatment with pexidartinib, if needed, patients can wait to start pexidartinib until after seeing a dietitian. If a referral to a dietitian is given, the prescriber should send information regarding the low-fat meal requirement to the dietitian in advance, specifying that “pexidartinib must be taken with a low-fat meal (approximately 11–14 g of total fat).” Examples of low-fat food options are available on the Turalio^®^ website [9]. It may also be worthwhile to ask patients to keep a food diary for a few days, which can be reviewed with the dietitian.

### Roles and responsibilities of the nursing team

Nurses will be involved in the initial discussion when a patient starts pexidartinib and they have an important role in patient education on the low-fat meal requirement, from the time the patient is prescribed pexidartinib until they begin taking the drug. Depending on the institution, different members of the nursing team, such as nurse educators, sarcoma nurses, and nurse navigators, may take on this role. As noted previously, nurses are often responsible for reviewing the low-fat meal requirement, initial education, and safety. Depending on site-specific processes, nurse navigators or other members of the nursing team (e.g., clinical or sarcoma nurses) will be responsible for meeting the requirements of the REMS and checking in with patients in between visits. Therefore, all members of the nursing team should be familiar with the low-fat meal requirement and should be able to follow-up with patients regarding any difficulties with understanding the low-fat meal requirement and planning pexidartinib dosing around their schedules. Patients should be encouraged to call in between visits with any questions or concerns regarding dosing or possible side effects.

Providers, nurses, and pharmacists will be involved in the initial discussion of starting pexidartinib. Before a patient starts taking pexidartinib, there should be a discussion about food; this should be ongoing while the patient is on pexidartinib. During check-ins, the patient’s labs will be reviewed and the nurse can use this time to ask about any side effects, difficulties with compliance, and any challenges with respect to the low-fat meal requirement. The nurse can then use this opportunity to explain the importance of taking pexidartinib with a low-fat meal and answer key questions related to the pexidartinib low-fat meal dosing regimen (Fig. 3). It will be important to ask patients, specifically what they are eating, and to point them to websites or applications that can help with meal design and planning.

### Pharmacist’s viewpoint

Pexidartinib is distributed through a specialty pharmacy. Patients need to understand that pexidartinib will be delivered to them from a specialty pharmacy and prescriptions for pexidartinib cannot be filled at a community pharmacy. Depending on the institutional model, pharmacists in both the clinic and the specialty pharmacy can assist with educating patients on pexidartinib administration. For this reason, an understanding of pexidartinib pharmacokinetics as it relates to meal content and timing is important.

#### Pharmacist-patient interaction

In our experience, a major concern of patients is how they are going to get their medication. The clinic pharmacist (or provider) should inform patients that the specialty pharmacy will contact them. After this point, pharmacists will review any medications to make sure there are no interactions. Pexidartinib interacts with moderate or strong CYP3A inhibitors, acid-reducing agents, and CYP3A substrates [3]. Pharmacists will then review the most common side effects, including what can be recognized and managed at home and when to call the pharmacy or clinic. The pharmacist will discuss follow-up appointments and their importance due to REMS and potential hepatotoxicity. When discussing with patients how to take the medication, a personalized approach is recommended. This does not need to be complex, however; it is likely easiest to determine how dosing will fit into the patient’s current meal schedule. If a patient has specific questions about diet or meal composition, referral to a dietitian should occur.

Patients are often concerned about what happens if they make a mistake with dosing. While it is important not to take a casual approach with respect to the timing of dosing (ie, they should be approximately 12 h apart), it is often necessary to work within the constraints of the patient’s meal schedule. The long half-life of pexidartinib means that there is some leeway with respect to dose timing. If a dose is missed, then the patient should be instructed to take the next dose at the scheduled time. If the patient takes pexidartinib without food, this is acceptable from time to time; the exposure to pexidartinib will be reduced in this instance, but it should not lead to unexpected side effects. Similarly, if pexidartinib is accidentally taken with a high-fat meal, total daily exposure will increase; such a scenario is not desirable but is not a cause for concern if it happens infrequently (ie, not a regular occurrence or pattern for a patient). It is important to emphasize to patients that pexidartinib should not be taken with a high-fat meal. Some patients may need to reduce their dosage due to side effects; compliance with their revised dosing should be carefully monitored and discussed with their provider.

Pharmacists are commonly involved in evaluating drug safety at the 1- or 2-week mark after starting pexidartinib by asking patients about adherence and side effects and reviewing lab values. Pharmacists will also ask about any new medications and whether a patient has any concerns. At this point, the importance of dosing pexidartinib with a low-fat meal and what constitutes a low-fat meal should be discussed with the patient again.

## Value of a dietitian

**Fig. 4:**
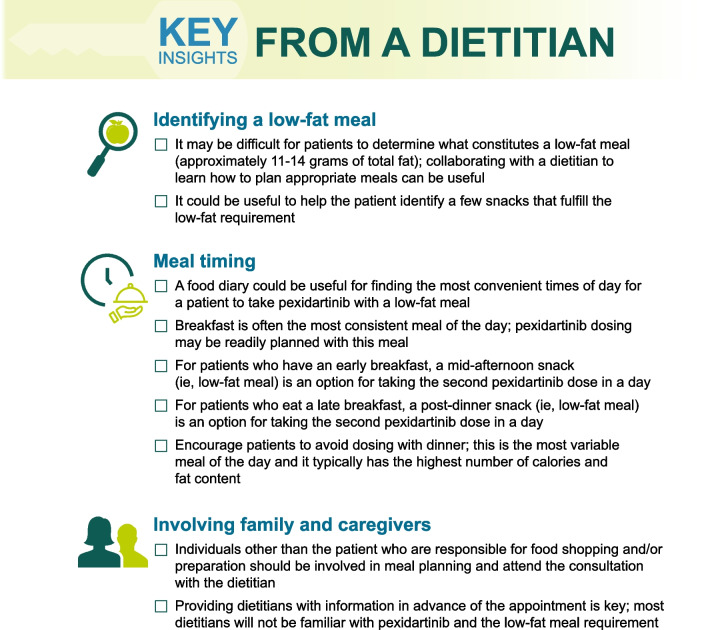
Dietitian insights on identifying a low-fat meal, meal timing, and involving family and caregivers

Dietitians are key resources to help patients understand nutritional content of meals and guide them to meals or snacks that fulfill the pexidartinib low-fat meal requirement. Although it would be ideal for every patient to consult a dietitian, this may not be possible due to insurance coverage, reimbursement limitations, delays in scheduling appointments, geographic location, and travel distance. Nevertheless, it may be helpful for patients to have an initial consult with a dietitian. A registered dietitian can be located online at the Academy of Nutrition and Dietetics Find a Nutrition Expert link: https://www.eatright.org/find-a-nutrition-expert. Figure 4 highlights key insights from a dietitian on identifying a low-fat meal, meal timing, and involving family and caregivers.

Overall, patients may find it difficult to determine if a certain meal is low fat; therefore, finding regular meals or snacks that fulfill the requirement will be important. Patients should be advised of applications and websites that can be helpful in determining whether a food or meal is low fat (e.g., https://fdc.nal.usda.gov, https://www.myfitnesspal.com, https://www.nutritionix.com, https://www.foodnoms.com, https://cronometer.com). Based on data from the PBPK model, when a patient takes their pexidartinib dose with a low-fat meal, they should wait ≥ 2 h before consuming a high-fat meal [7]. Similarly, when a high-fat meal is consumed, patients should wait ≥ 3 h before taking their dose of pexidartinib with a low-fat meal [7]. The PBPK modeling study also demonstrated that fat content, not total number of calories, was the most important factor influencing pexidartinib pharmacokinetics [7]. These factors should be considered when timing pexidartinib dose administration with respect to meals.

## Conclusions

The pexidartinib dosing change was proposed due to the known effects food has on the absorption of pexidartinib and the potential for drug overexposure. Additionally, this dosing change was designed to improve patient compliance. The prescribing physician, nurse practitioner, or other provider relies on the nursing team, pharmacists, and dietitians to support patients in the transition to the new pexidartinib dosing regimen that includes the low-fat meal requirement. Although it would be useful for all patients to see a dietitian, this may not be possible due to limitations with insurance or accessibility. The example educational resources and key insights from a dietitian can be used within the multidisciplinary team to assist with patient compliance as they transition to the new dosing regimen.

## Data Availability

All relevant data are provided within the manuscript.

## Abbreviations

ALT: Alanine aminotransferase
AST: Aspartate aminotransferase
AUC_inf_: Area under the plasma concentration–time curve from time 0 extrapolated to infinity
AUC_last_: Area under the plasma concentration–time curve from time 0 to the last quantifiable concentration
C_max_: Maximum plasma concentration
FDA: US Food and Drug Administration
PBPK: Physiologically based pharmacokinetic
RECIST: Response Evaluation Criteria in Solid Tumors version 1.1
REMS: Risk Evaluation and Mitigation Strategy
TGCT: Tenosynovial giant cell tumor
T_max_: Time to maximum plasma concentration
TVS: Tumor volume score
